# Nut Consumption and Lung Cancer Risk: Is Linoleic Acid the Secret Weapon?

**DOI:** 10.22037/ijpr.2020.1101216

**Published:** 2020

**Authors:** Raffaella Mormile

**Affiliations:** *Division of Pediatrics and Neonatology, San Giuseppe Moscati Hospital, Aversa, Italy.*

Nut consumption has been shown to be inversely related to lung cancer, and associations have been reported to be independent of cigarettes smoking and other known risk factors (1). The nuts are natural foods rich in unsaturated fatty acids (2, 3). Almost one-half of the total fat content consists of monounsaturated acids (MUFA) and polyunsaturated acids (PUFA) such as linoleic acid, which is an omega-6 PUFA (3). Healthy fats in nuts have long been observed to contribute to beneficial effects of the frequent nut intake against a number of pathological conditions such as coronary artery disease and diabetes, in epidemiological studies (2, 3). Nuts represent one of the major dietary sources of linoleic acid (2, 3). Most nuts contain substantial amount of linoleic acid (2). Current evidence links this compound with cancer risk and progression (4). Linoleic acid intake has been inversely connected with telomere length (4). Telomeres are localized in the physical ends of eukaryotic chromosomes (3). They are critical in maintaining the structural integrity of the genome and in protecting chromosome from degradation preventing the loss of genetic information (2). Telomeres experience erosion with each cycle of replication, and this shortening may relate to cellular senescence or apoptosis and cell death (3, 4). Telomere length in humans is primarily regulated by three major components including human telomerase reverse transcriptase (hTERT) (4). This enzyme is highly expressed in cancer cells, but not in normal cells (4). Malignant cells show pronounced activation of telomerase, which adds telomeric repeats to the end of replicating chromosomes in order to prevent telomere shortening, and it subsequently leads to immortal cell characteristics and tumorigenesis (4). Interestingly linoleic acid has been detected to potently suppress the telomerase activity via down-regulation of hTERT in transcription and translation (4). Recently, it has been suggested that longer telomere length in peripheral blood cells is a likely risk factor for lung cancer (5). Thus, I speculate that linoleic acid supplementation may represent the secret weapon of nuts in lung cancer prevention and survival taking into account the up-regulation of hTERT expression underlying longer telomere length in lung cancer. I advise that linoleic acid down-regulates telomerase activity avoiding telomere lengthening and consequently, inhibiting lung cancer cells proliferation. I propose to promote country-specific nutritional approaches to the prevention of lung cancer through linoleic acid-rich foods taking into account differences in linoleic acid intake by race/ethnicity. Proteomics analysis and metabolomic profiling studies might facilitate an understanding of changes in specific proteins and metabolites in response to nut consumption and linoleic acid intake in lung cancer. Identifying the molecular mechanisms through which nut consumption and linoleic acid intake affect lung cancer risk may provide an opportunity to implement focused dietary interventions in order to develop personalized diets for cancer prevention. More trials are required for approving this hypothesis about nuts in lung cancer.
